# ‘Harm? I don’t think so!’: medical overuse from the perspective of allied health professionals in Germany – a qualitative study

**DOI:** 10.1136/bmjopen-2025-102991

**Published:** 2025-11-04

**Authors:** Benedikt Stelzner, Laura Rink, Thomas Kühlein, Maria Sebastião

**Affiliations:** 1 Institute of General Practice, Friedrich-Alexander-Universität Erlangen-Nürnberg, Erlangen, Germany

**Keywords:** QUALITATIVE RESEARCH, Decision Making, Health Services, Quality in health care

## Abstract

**Abstract:**

**Objectives:**

Medical overuse is a well-documented increasing issue, primarily examined in the context of physicians. Previous research has also identified unnecessary services involving allied health professionals (AHPs). The objectives of our study were to explore: (1) To what extent are physiotherapists (PT), occupational therapists (OT) or speech and language therapists (SLT) familiar with the phenomenon of medical overuse?, (2) What drivers do PTs, OTs and SLTs suspect?, (3) What are the consequences of medical overuse? and (4) What measures can be taken to reduce medical overuse?

**Design:**

This study used a qualitative descriptive design and applied qualitative content analysis to explore the AHPs’ point of view. A qualitative content analysis using a deductive–inductive approach was conducted. After coding half of the interviews, no further categories were added, indicating data saturation.

**Setting:**

Bavaria, Germany.

**Participants:**

14 AHPs, mostly female.

**Results:**

AHPs struggled to define overuse. To them, underuse was perceived as a much more pressing issue. AHPs identified structural, economic, physician and patient-driven factors. They did not see themselves as part of the problem of medical overuse and assumed that their treatment, even without indication, has little to no disadvantage for patients. AHPs found it difficult to derive specific solutions; they named terminating unnecessary therapies and healthcare system reforms.

**Conclusions:**

AHPs lacked initial awareness of medical overuse, highlighting the need for education and broader research.

**STRENGTHS AND LIMITATIONS OF THIS STUDY**

- Due to the qualitative data collection and analysis, new topics could emerge.
- Sampling was data driven and we reached data saturation.
- The study did not originally aim to explore medical underuse, so findings in this regard may lack systematic depth.
- Multiple steps were taken to ensure the quality of this study, such as interviewer training, pilot testing of the interview guide, keeping a research diary and discussing codes in the team and with other researchers.

## Background

 Medical overuse occurs when ‘a healthcare service is provided under circumstances in which its potential for harm exceeds the possible benefit’.^1^ Beyond contributing to physical and psychological harm to patients,^2^ medical overuse can also cause increased healthcare costs^3^ and therefore poses additional strain on the healthcare system.^4^ The negative impacts of medical overuse can be illustrated using the example of non-specific lower back pain. About 50% of the German population experienced lower back pain within the last 12 months.^5^ Despite the recommendation that imaging procedures should be avoided in non-specific lower back pain during the first weeks, they are still being performed and the numbers have even increased in the last years.^6^ Apart from the unnecessary costs, this is also problematic as it increases overdiagnosis^7^ and is associated with poorer health outcomes in patients.^8^

Surveys among patients have already been conducted in different countries and showed that they are little aware of the problem of medical overuse.^9^,^14^ Studies have shown that general practitioners (GPs) knew the issue.^15 16^ Not all GPs acknowledged the occurrence of medical overuse in their own practice.^16 17^ The main drivers identified by GPs were patient expectations, the absence of a primary care system and defensive medicine.^18^,^20^

As opposed to patients and physicians, allied health professionals’ (AHPs) points of view have rarely been considered in studies concerning medical overuse. Research suggests that unnecessary measures are also being provided in non-medical professions such as physiotherapy (PT).^21^ For example, PT is being prescribed for non-specific lower back pain, although within 14 days, there are no clinically relevant differences between patients receiving PT compared with a group without it.^22^ In up to 25%, massages can even increase the pain.^23^ Medical overuse might also occur in other therapy professions such as occupational therapy (OT) or speech and language therapy (SLT), which have been unrepresented in studies so far.

The objectives of our study were to explore: (1) To what extent are PTs, OTs and SLTs familiar with the phenomenon of medical overuse?, (2) What drivers do PTs, OTs and SLTs suspect?, (3) What are the consequences of medical overuse? and (4) What measures can be taken to reduce medical overuse?

## Methods

### Study design

This study used a qualitative descriptive design and applied qualitative content analysis to explore the AHPs’ point of view. From June 2022 to February 2023, semi-structured single interviews with AHPs were conducted.

### Recruitment and setting

At first, BS contacted AHPs in Bavaria, Germany randomly and informed them about the study via flyer and/or email. AHPs could further spread the information. AHPs expressing interest subsequently received the study information, the data protection declaration, a consent form for signature and a brief socio-demographic survey for completion. We planned to include not only one kind of AHP but PTs, OTs and SLTs instead, to better capture system-level perceptions of medical overuse. Our aim was to capture how AHPs perceive medical overuse in routine practice, rather than to elicit only expert definitions. Inclusion of early-career AHPs was intentional as they constitute a substantial share of the German workforce and are most likely to have academic training (and exposure to evidence-based practice/guidelines). After the first interviews, recruitment was carried out iteratively, allowing for targeted engagement with specific professional groups following data collection and initial analysis. For example, the first three therapists interviewed had completed training, so we specifically looked for therapists with degrees. Another example is age of the interviewees: as the first interviewees were under 30 years of age, we thereafter sought out therapists who were older than 40 years as a contrast (interviews 6, 7, 8). All interested AHPs could take part in the study. Participants were informed about the goal of the study and gave written informed consent. The interviewer (BS, male, student of human medicine) and the AHPs did not know each other beforehand.

### Data collection

The project team developed the interview guide which comprised four topic sections: (1) clinical decision-making based on a case vignette, (2) prior knowledge and experience of medical overuse, (3) drivers and consequences of overuse and (4) solutions for reducing medical overuse (online supplemental file 1). The interview guide underwent a pre-testing phase involving two AHPs. BS was trained in qualitative data collection by TK (male, Chair of Institute, GP) and MS (female, senior researcher, SLT/Public Health). BS conducted all interviews (n=14, between 40 and 60 min, average 54 min in length) via video calls using the software Zoom. Except for the participants, there was no one else present during data collection. The sessions were digitally recorded, subsequently transcribed verbatim and pseudonymised using 4 software. Interviews did not need to be repeated. Transcripts were not provided to participants.

### Data analysis

BS conducted a qualitative content analysis using a deductive–inductive approach (with the software MAXQDA Plus 2020). Initially, categories were established based on the interview guide (perceptions of medical overuse, drivers, consequences of medical overuse and measures to reduce overuse), which were then systematically applied to the data (deductive coding). The deductive frame comprised 21 subcategories in total (10 first-order and 11 second-order).

Throughout the analysis process, the category system was iteratively expanded through inductive coding (eg, underuse, insufficient knowledge, therapist–patient relationship, lack of communication; 10 new subcategories emerged). All categories were continuously discussed by the project team. We monitored data saturation continuously during coding. After coding half of the interviews, no further categories were added, indicating data saturation. All data were coded using the final category system. Participants did not provide feedback on the findings. BS maintained a research diary to reflect his involvement and potential impact throughout the research process. The reporting of the study follows the ‘Consolidated criteria for reporting qualitative research’.^24^

### Quality assurance

We took multiple steps to assure the quality of this study: the interviewer had training in qualitative interviewing by two senior researchers (LR and MS), the interview guide was piloted with two AHPs and refined accordingly, data triangulation took place through interviews with different AHPs (heterogeneity across profession, age, gender, qualification and setting), after each interview field notes were made (eg, disturbances during interview, first ideas for further recruitment and/or data analysis), the research diary was not only kept the entire research process, but also repeatedly incorporated into data collection and analysis. To strengthen dependability and confirmability, MS reviewed coding and discrepancies were discussed to consensus. We also conducted a qualitative research workshop to code and discuss codes with other researchers.

### Patient and public involvement

Patients and/or the public were not involved in the design, conduct, reporting or dissemination plans of this research.

## Results

### Study population

Of the 14 AHPs interviewed, most were female (n=10) and were employed (table 1).

**Table 1:** Sample characteristics

		n (%)
Sex	Male Female	4 (29) 10 (71)
Age groups	18–29 years 30–39 years 40–49 years 50–65 years	7 (50) 4 (29) 2 (14) 1 (7)
Profession	PT OT SLT	7 (50) 4 (29) 3 (21)
Working experience in years	0–2 3–5 6–10 >11	1 (7) 6 (43) 3 (21) 4 (29)
Employment status	Self-employed Employed	5 (36) 9 (64)
Number of AHPs in practice	1–3 4–10 >11	2 (15) 9 (64) 3 (21)
Professional education	Vocational training Bachelor Master Doctorate	11 (79) 3 (21) 0 (0) 0 (0)
Member of a professional organisation	Yes No	4 (29) 10 (71)

AHP, allied health professional; OT, occupational therapy; PT, physiotherapy; SLT, speech and language therapy.

### Therapists were not familiar with medical overuse

Therapists found it challenging to define medical overuse: ‘So, I haven’t really dealt with the term. […] No idea’ (SLT03). They associated medical overuse with a structural surplus of personnel rather than medically unnecessary services: ‘So, for me, medical overuse would mean that we have too many AHPs, too many practices in one location’ (PT04). Medical overuse was often associated with incorrect treatment due to lack of knowledge (OT03), insufficient preparation (SLT01) or lacking resources elsewhere. For example, social needs are met through therapy, and visiting the AHPs has become ‘a very, very important cornerstone of life’ (SLT01). It is not the treatment itself that is the focus, but rather the social component (OT03). Those who could define medical overuse indicated that they have engaged with it as preparation for the interview (eg, OT4).

### Examples of medical overuse

The AHPs were able to mention specific unnecessary treatments. However, it was noticeable that this only became possible during the course of the interview, after a certain level of engagement with the topic had already occurred. Examples of medical overuse were (1) within their profession (‘Thermal treatments […] that’s absolutely pointless’ (OT04)). Especially in SLT, it was challenging for participants to name examples: ‘This is the only area that comes to mind […] children with bilingualism. First of all, to differentiate, does the child need therapy or language support? That’s a fine line’ (SLT02). (2) Within other allied healthcare professions (eg, massages for certain problems), and (3) other professions/medical areas. Here, AHPs found it easier to provide examples, such as surgeries, or excessive prescription of medications (eg, antibiotic administration for influenza-like infections or polypharmacy in older individuals) (PT03).

### Underuse is a more urgent problem

In the interviews, many AHPs spontaneously contrasted medical overuse with underuse (eg, long waiting lists, workforce shortages) and framed underuse as the more pressing day-to-day problem: ‘Everyone could not only double themselves but triple, and we would still have plenty to do’ (OT01). For them, patients also receive too few prescriptions for therapy: ‘You just have to say that you have to kind of fight for prescriptions for therapy’ (SLT02).

### Drivers

**Figure 1:**
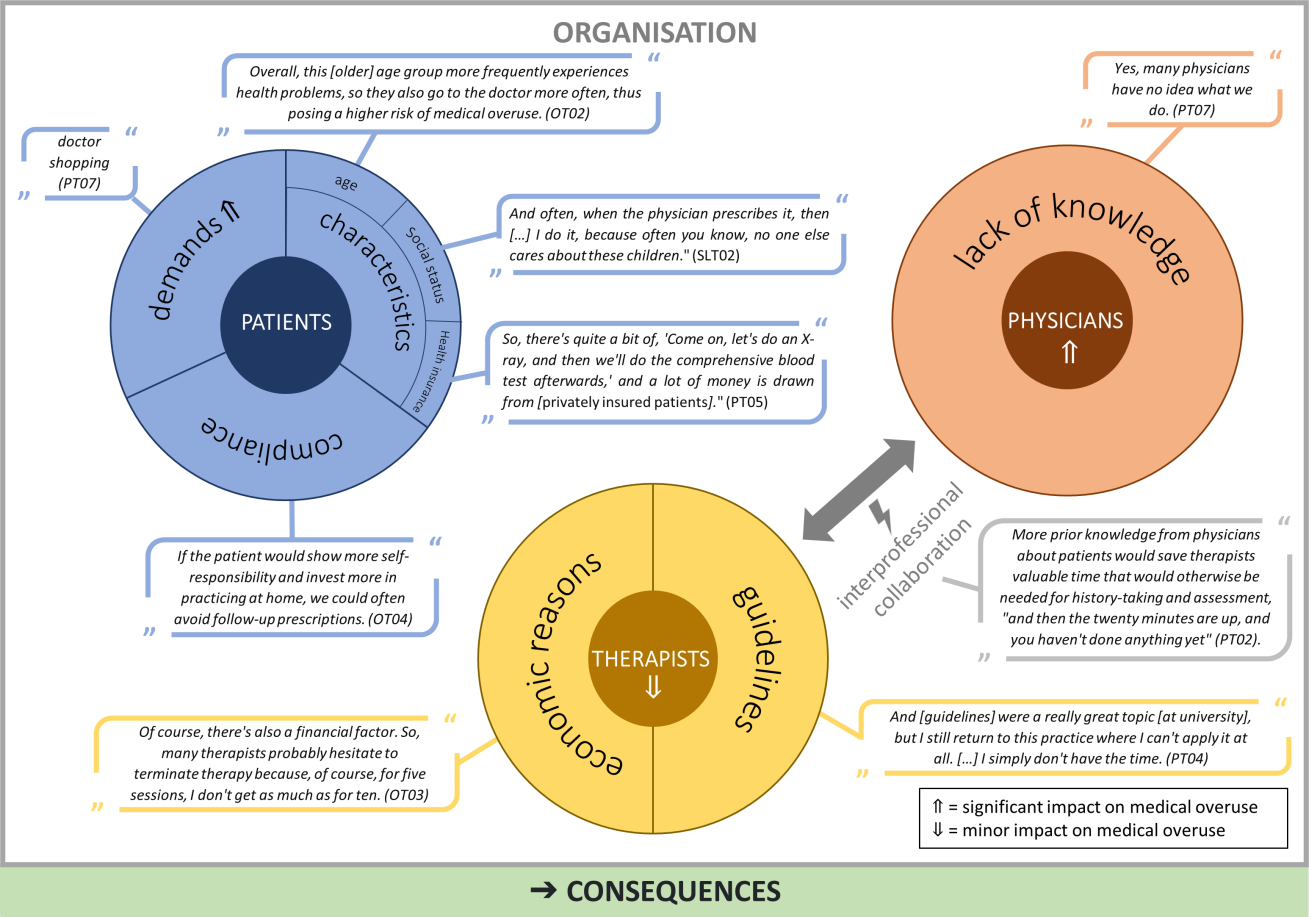
Drivers of medical overuse. OT, occupational therapy; PT, occupational therapy; SLT, speech and language therapy.

AHPs mentioned drivers of medical overuse and identified physicians as the main driver of medical overuse but not concerning PT, OT or SLT, where the AHPs described only a few cases in which physicians unnecessarily prescribed therapy (PT02) (figure 1). In their opinion, physicians drive medical overuse in their own field. In general, AHPs did not see themselves as part of the problem of medical overuse. However, during the course of the interviews, some respondents reflected more on their role, for example, whether the problem of waiting lists is self-inflicted, as patients continue to be treated despite questionable effects (PT03). However, they described little to no action taken to refrain from treating patients to free up resources.

### Organisational structures

The drivers are embedded in organisational structures. Regular PT was referred to as a kind of ‘trend’ (PT01), something many people indulge in after a stressful day at work. This phenomenon is also observed among young age groups as parents send their children for minor issues that, according to the AHPs, do not require treatment. ‘Well, my child absolutely must go to the PT’ (PT01). They attributed this to societal changes: ‘a reflection of our time. Always faster’ (PT07). Furthermore, the current healthcare system provides false financial incentives, leading to the offering of unnecessary services to patients (OT02).

### Consequences of medical overuse

The AHPs assumed that their treatment, even without indication, has little to no disadvantage for patients: ‘Harm? I don’t think so!’ (OT03). They did not address iatrogenic fixation or increased pain, for example, in acute back pain, or stigmatisation. Medical overuse leads to higher healthcare expenditures. For the AHPs, this issue plays a secondary role, even though there is general awareness of the topic: ‘There are always costs that rise’ (PT04).

### Measures to reduce medical overuse

The statements to reduce medical overuse were brief and indicated that AHPs found it difficult to derive specific measures. They named the responsibility to terminate therapies when appropriate (OT03) and direct access to therapy (SLT02). Campaigns like Choosing Wisely were largely unknown. However, after explaining them, some AHPs wished for initiatives similar to Choosing Wisely (PT01). They also wished for ‘a revolution’ *(*PT04) of the healthcare system as ‘Reducing medical overuse means getting rid of these case rates, so you don’t have the pressure. You have to get the money by offering patients this and that or not offering’ (OT02).

## Discussion

AHPs were mostly unfamiliar with the concept of medical overuse. They associated medical overuse with too many AHPs, low quality of therapy due to lack of knowledge or insufficient preparation and lacking resources elsewhere. Underuse was a more pressing topic to them. They saw physicians as the main drivers and themselves with little opportunity to influence medical overuse. The lack of interprofessional communication worsens the situation. AHPs named age, private health insurance, social status and non-compliant patients as driving factors. They also described patients as demanding and getting treatment as a new form of lifestyle. Mostly, economic reasons were mentioned on their side, and overall, the AHPs believed that there are false financial incentives. AHPs found it hard to propose solutions and named direct access to them as a way to mitigate medical use. They did not know initiatives such as Choosing Wisely beforehand but supported the idea.

The lack of knowledge about medical overuse was also evident in other studies.^14 25^ Participants’ focus on underuse (eg, long waiting lists) helps explain why AHPs perceived medical overuse as less immediate and, at times, harder to recognise.

The dependency on the prescription from physicians could explain why AHPs saw themselves as having little responsibility for medical overuse. AHPs saw the main driver of medical overuse in physicians. Patients’ demands seem to be another critical driver of medical overuse which was stated in previous studies.^14 15 26 27^ It was not clear whether the patients always verbalise these demands or whether they are assumed by physicians/therapists. Other studies have shown that physicians sometimes act based on assumed demands.^28^ The phenomenon of blaming others could also be found in physicians and patients.^14 16^

AHPs also believed that contact with the healthcare system itself already raises the risk of medical overuse which was also mentioned by patients in Germany.^14^ Routines are another factor that drives medical overuse as they do not require a conscious decision-making.^29 30^ This point was not mentioned directly by the AHPs, but it became apparent that the AHPs rarely deviated from the treatment plan, for example, by ending the treatment earlier than prescribed.

AHPs saw little to no harm in therapy even without indication. It can be assumed that AHPs, like patients and physicians,^31 32^ overestimate the benefits and underestimate the harms of interventions.

Studies have shown incorrect beliefs about low back pain treatment among PTs.^33 34^ In an international review, between 48% and 70% of PTs could critically appraise research papers.^35^ Training in evidence-based therapy may vary greatly in Germany due to different training paths. It is possible to start treating patients after a 3-year vocational training or to continue studying at universities of applied health sciences to receive a Bachelor’s or Master’s degree. A survey of 1361 German physiotherapists revealed that only one-third were familiar with the national guideline on unspecific low back pain. Longer professional experience could have a negative impact on treatment choices.^36^ Academically trained PTs perform slightly better in diagnostics,^37^ but the overall quality of decision-making remains below the ideal level. Learning to understand what evidence is and how it should be applied in practice should be trained intensively during all training stages. The lack of knowledge about medical overuse demonstrates the importance of sensitising AHPs to the issue so that they can inform and educate patients in the long run—particularly as they often have more time with patients than physicians. However, further training was not mentioned by the AHPs as a solution.

Overall, proposed solutions were pragmatic but sparse: improving interprofessional communication which was also wished for by German GPs^26^ and system-level reforms. A complete electronic medical record—which was introduced for all statutory health insured patients in Germany in 2025—might support this need. In 2024, a new regulation was introduced for selected indications, allowing OTs and PTs to independently determine the type and amount of treatment provided (‘open prescription’). With this change, financial responsibility was also transferred to the therapists: beyond a certain number of therapy sessions, financial deductions must be accepted. This regulation is not equivalent to direct access as in other countries which was mentioned as a solution. However, it grants AHPs more therapeutic freedom and responsibility. The impact of this regulation on existing care shortages and therapists’ role perceptions is yet to be determined. In other studies, direct access to physical therapy showed promising results on managing musculoskeletal disorders regarding, for instance, healthcare costs.^38^,^40^

Clear recommendations (eg, Choosing Wisely-style lists) were viewed favourably. An example could be the living guideline ‘Protection against the Overuse and Underuse of Health Care’ published by The German College of General Practitioners and Family Physicians (DEGAM) which compiles recommendations for protection against overuse and underuse. A survey among German GPs showed that the majority of GPs found the guideline helpful and wished for further recommendations.^26^ Choosing Wisely Brazil, for example, developed a top five low-value-list for musculoskeletal physical therapy.^41^ Kharel *et al* showed that PTs were more willing to follow detailed and positively framed recommendations or if evidence-based alternatives are given.^42^ These findings can help formulate recommendations for German AHPs as well. However, lists of recommendations on their own are unlikely to change practice^21^ as a wide range of system drivers promote medical overuse. This was also seen by AHPs who wished for changes to the reimbursement system. They rated false financial incentives as an important driver of medical overuse, which is in line with previous studies.^16^

### Limitations

AHPs voluntarily and without remuneration participated in the present study which may have led to a selection bias. Despite the small sample, we were able to recruit AHPs from all three disciplines. However, we were not able to include AHPs with a Master’s or doctorate degree. We deliberately did not exclude the participants that were unfamiliar with medical overuse, as this is a key finding and is consistent with findings from other professions. Across professions, answers were largely homogeneous. Unequal numbers in the sample across professions reflect saturation rather than bias—SLT yielded no substantially new insights after interview three, whereas additional PT interviews added nuance. Given the shared dependency on medical referral across these professions, common system drivers outweighed profession-specific differences. The study did not set out to examine medical underuse. However, the qualitative descriptive study design allowed for new topics to emerge such as underuse. These results were participant-led and reported to contextualise perceptions of medical overuse.

### Implications for practice

- AHPs need further training to better understand the benefits and harms of their treatment.
- AHPs should be included in guideline development to ensure greater practicability.
- Change processes should begin where AHPs already show an emerging awareness of problems after study participation (eg, fully using prescriptions even when they are no longer necessary). However, changes should not result in income loss in order to promote acceptance.

### Implications for future research

- Based on our results, a quantitative study with a focus on practical solutions may follow.
- Our study did not set out to examine medical underuse; results regarding underuse were participant-led and reported to contextualise perceptions of medical overuse. Future work should further investigate medical underuse with dedicated aims, sampling and measures.
- To our knowledge, research primarily focuses on PTs. Future studies should also focus on OTs and SLTs.

## Conclusion

Our results showed on the one hand that AHPs were rather unfamiliar with the concept of medical overuse, suggesting that more awareness raising is needed. On the other hand, the AHPs developed an understanding of the issue, showing that asking or better education can trigger reflection. The participants found it easier to name medical overuse within PT (eg, massages). To our knowledge, research primarily focuses on PTs. Future studies should also focus on OTs and SLTs. The study did not set out to examine medical underuse; results regarding underuse were participant-led and reported to contextualise perceptions of medical overuse. Future work should further investigate medical underuse with dedicated aims, sampling and measures.

## Supplementary material

10.1136/bmjopen-2025-102991 online supplemental file 1

## Data Availability

Data are available upon reasonable request.

## References

[1] Chassin MR, Galvin RW (1998). The urgent need to improve health care quality. Institute of Medicine National Roundtable on Health Care Quality. JAMA.

[2] Korenstein D, Chimonas S, Barrow B (2018). Development of a Conceptual Map of Negative Consequences for Patients of Overuse of Medical Tests and Treatments. JAMA Intern Med.

[3] Schwartz AL, Landon BE, Elshaug AG (2014). Measuring low-value care in Medicare. JAMA Intern Med.

[4] Kühlein T, Macdonald H, Kramer B (2023). Overdiagnosis and too much medicine in a world of crises. BMJ.

[5] Lippe E, Krause L, Porst M (2021). Prävalenz von Rücken- und Nackenschmerzen in Deutschland. Ergebnisse der Krankheitslast-Studie BURDEN 2020. Journal of Health Monitoring.

[6] Downie A, Hancock M, Jenkins H (2020). How common is imaging for low back pain in primary and emergency care? Systematic review and meta-analysis of over 4 million imaging requests across 21 years. Br J Sports Med.

[7] Brinjikji W, Luetmer PH, Comstock B (2015). Systematic Literature Review of Imaging Features of Spinal Degeneration in Asymptomatic Populations. AJNR Am J Neuroradiol.

[8] Webster BS, Cifuentes M (2010). Relationship of early magnetic resonance imaging for work-related acute low back pain with disability and medical utilization outcomes. J Occup Environ Med.

[9] Ghanouni A, Renzi C, Waller J (2018). Improving public understanding of “overdiagnosis” in England: a population survey assessing familiarity with possible terms for labelling the concept and perceptions of appropriate terminology. BMJ Open.

[10] Moynihan R, Nickel B, Hersch J (2015). Public Opinions about Overdiagnosis: A National Community Survey. PLoS One.

[11] Moynihan R, Nickel B, Hersch J (2015). What do you think overdiagnosis means? A qualitative analysis of responses from a national community survey of Australians. BMJ Open.

[12] Park SH, Lee B, Lee S (2015). A qualitative study of women’s views on overdiagnosis and screening for thyroid cancer in Korea. BMC Cancer.

[13] Verkerk EW, Boekkooi JAH, Pels EGM (2023). Exploring patients’ perceptions of low-value care: An interview study. Patient Educ Couns.

[14] Sebastião M, Pesch J, Kühlein T (2024). “The health care system is more like a business’-medical overuse from the patients” perspective in Germany: a qualitative study. BMJ Open.

[15] Kool RB, Verkerk EW, Winnemuller LJ (2020). Identifying and de-implementing low-value care in primary care: the GP’s perspective-a cross-sectional survey. BMJ Open.

[16] Alber K, Kuehlein T, Schedlbauer A (2017). Medical overuse and quaternary prevention in primary care - A qualitative study with general practitioners. BMC Fam Pract.

[17] Hambrock U (2019). Erfahrungen Mit Überversorgung [Experience with Medical Overuse].

[18] Mira JJ, Carrillo I, Silvestre C (2018). Drivers and strategies for avoiding overuse. A cross-sectional study to explore the experience of Spanish primary care providers handling uncertainty and patients’ requests. BMJ Open.

[19] van Dulmen SA, Naaktgeboren CA, Heus P (2020). Barriers and facilitators to reduce low-value care: a qualitative evidence synthesis. BMJ Open.

[20] Pausch M, Schedlbauer A, Weiss M (2020). Is it really always only the others who are to blame? GP’s view on medical overuse. A questionnaire study. PLoS One.

[21] Traeger AC, Moynihan RN, Maher CG (2017). Wise choices: making physiotherapy care more valuable. J Physiother.

[22] Zadro J, O’Keeffe M, Maher C (2019). Do physical therapists follow evidence-based guidelines when managing musculoskeletal conditions? Systematic review. BMJ Open.

[23] Furlan AD, Giraldo M, Baskwill A (2015). Massage for low-back pain. Cochrane Database Syst Rev.

[24] Tong A, Sainsbury P, Craig J (2007). Consolidated criteria for reporting qualitative research (COREQ): a 32-item checklist for interviews and focus groups. Int J Qual Health Care.

[25] Nürnberger C, Kühlein T, Hueber S (2024). What do people know and think about medical overuse? an online questionnaire study in Germany. PLoS One.

[26] Warkentin L, Scherer M, Kühlein T (2024). Evaluation of the German living guideline “Protection against the Overuse and Underuse of Health Care” - an online survey among German GPs. *BMC Prim Care*.

[27] Fraser GRL, Lambooij MS, van Exel J (2024). Factors associated with patients’ demand for low-value care: a scoping review. BMC Health Serv Res.

[28] Gogineni K, Shuman KL, Chinn D (2015). Patient Demands and Requests for Cancer Tests and Treatments. JAMA Oncol.

[29] Nilsen P, Roback K, Broström A (2012). Creatures of habit: accounting for the role of habit in implementation research on clinical behaviour change. Implement Sci.

[30] Potthoff S, Rasul O, Sniehotta FF (2019). The relationship between habit and healthcare professional behaviour in clinical practice: a systematic review and meta-analysis. Health Psychol Rev.

[31] Hoffmann TC, Del Mar C (2015). Patients’ expectations of the benefits and harms of treatments, screening, and tests: a systematic review. JAMA Intern Med.

[32] Hoffmann TC, Del Mar C (2017). Clinicians’ Expectations of the Benefits and Harms of Treatments, Screening, and Tests: A Systematic Review. JAMA Intern Med.

[33] Christe G, Nzamba J, Desarzens L (2021). Physiotherapists’ attitudes and beliefs about low back pain influence their clinical decisions and advice. Musculoskelet Sci Pract.

[34] Derghazarian T, Simmonds MJ (2011). Management of low back pain by physical therapists in quebec: how are we doing?. Physiother Can.

[35] da Silva TM, Costa L da C, Garcia AN (2015). What do physical therapists think about evidence-based practice? A systematic review. Man Ther.

[36] Kühn L, Rosen D, Reiter NL (2024). Appropriateness of exercise therapy delivery in chronic low back pain management: cross-sectional online survey of physiotherapy practice in Germany. BMC Musculoskelet Disord.

[37] Konrad R, Geraedts M (2018). Case-oriented selection of investigation methods in direct access: A comparison between physiotherapy trainees at professional colleges and in bachelor’s study courses. GMS J Med Educ.

[38] Babatunde OO, Bishop A, Cottrell E (2020). A systematic review and evidence synthesis of non-medical triage, self-referral and direct access services for patients with musculoskeletal pain. PLoS One.

[39] Demont A, Bourmaud A, Kechichian A (2021). The impact of direct access physiotherapy compared to primary care physician led usual care for patients with musculoskeletal disorders: a systematic review of the literature. Disabil Rehabil.

[40] Clark B, Clark L, Showalter C (2022). A call to action: direct access to physical therapy is highly successful in the US military. When will professional bodies, legislatures, and payors provide the same advantages to all US civilian physical therapists?. J Man Manip Ther.

[41] Reis FJJ, Meziat-Filho N, Soares RJ (2021). Choosing Wisely Brazil: top 5 low-value practices that should be avoided in musculoskeletal physical therapy. Physiotherapy.

[42] Kharel P, Zadro JR, Ferreira G (2023). Can language enhance physical therapists’ willingness to follow Choosing Wisely recommendations? A best-worst scaling study. Braz J Phys Ther.

