# Supplementary material for: ‘Harm? I don’t think so!’: medical overuse from the perspective of allied health professionals in Germany – a qualitative study
Source: BMJ Open. 2025 Nov 4;15(11):e102991. doi: 10.1136/bmjopen-2025-102991 (PMC12587934; doi:10.1136/bmjopen-2025-102991)
Supplement: online supplemental file 1 [file bmjopen-15-11-s001.docx]

**Interview guide for the study „Perception of medical overuse from the perspective of therapists – a qualitative study“**

1. **Beginning**

Thank you very much for taking part in the interview. Today we would like to talk about the topic of medical overuse. First, I would like to get to know your everyday working life. Can you tell me about your working day today/yesterday?

- How was your day
- Was it a typical day for you?
- Was something different?
- Which patients do you typically treat? Does the practice treat a focus group of patients? What about additional private services?

1. **Clinical reasoning**

**Case physio therapists:** A young lady presents to your practice with new, uncomplicated back pain. She reports that her family doctor was unable to find a specific cause and ruled out any signs of a serious course. The prescription of remedies says “lumbar spine syndrome”. After two sessions the patient is free of symptoms. How would you proceed?

**Case occupational therapists:** A 40-year-old patient receives a prescription of remedies for occupational therapy from his orthopedic surgeon due to a fracture of the wrist. Halfway through the treatment, there is a significant improvement in mobility and muscle strength has also been restored. How would you proceed?

**Case speech therapists:** A neurologist prescribes a prescription of remedies for speech therapy treatment (10 prescription of remedies) to an elderly woman with peripheral facial paralysis. After seven appointments you will achieve a complete recovery of neurological functions. How would you proceed?

- How do you generally make decisions about your therapy? Do you determine this based on specific parameters? If so, where did you get these (guidelines, experience)? (Sketching a patient)
- When would you end therapy prematurely? Based on what aspects do you decide? Do you decide this alone, after consulting with the practice or with the patient? (Everything must be “normal” at the end of the treatment?)
- At what point do you recommend a follow-up prescription of remedies to a patient?
- Could further treatments also harm the patient?
- You are not entirely sure about your treatment decision. How do you deal with uncertainty?
- How do you deal with patients who have different opinions about the therapy than you? (pre-informed patient)

1. **Previous knowledge about medical overuse**

What do you understand by medical overuse?

What do you think medical overuse could lead to?

| **Definition of medical overuse**  Medical overuse, i.e. care with services that are not indicated, or with services without a sufficiently proven net benefit (medical overcare) or with services that have only a small benefit that no longer justifies the costs or are provided in an inefficient, i.e. uneconomic form (economic overcare).  Medical overuse: Care without sufficiently proven net benefits.  Economic overuse: Supply with only a small benefit that no longer justifies the costs (inefficiency) |
| --- |

- As you read this definition, what do you mean about medical overuse now? (Comparison with previous statement)
- Have you heard about unnecessary medical treatments in the healthcare system?
- How could medical overuse be applied to your work? What shapes can be found here?
- How would you in contrast to medical overuse define
  - Underuse
  - incorrect care?
- Which do you think poses the biggest problem?
- Do you think overuse is an issue for all therapists? If not, who not and why?
- What is your patient’s awareness of unnecessary treatments?
- Does it happen that therapies are questioned? (From yourself, patient, doctor, professional society?
- To what extent might overuse occur differently in different age groups?

1. **Choosing wisely**

A general aim of “Choosing Wisely” is to sensitize professional groups to the issue of overuse and to promote public discourse about unnecessary treatments . The focus is primarily on treatments, that

- Be carried out in large numbers
- Their benefit for the patient is inadequate
- Produce a lot of costs
- Too complex to implement in practice

*Questions about the drivers und consequences of medical overuse*

- How do you see this campaign? Should therapists also be involved in the Choosing Wisely initiative to reduce overuse?
- Why do you think so many unnecessary treatments are carried out? (Patient request, therapist error, training, doctor, convenience)
  - How do you deal with treatments that bring little benefit to your patients?
  - Do you inform your patients in advance about possible risks and limitations of treatments?
- What about treatments that fulfil or partially fulfil the above criteria? Can you name any? In your sector?
  - Could you specifically name such treatments? If yes, =>
  - To what extent can you measure the success of a therapy for yourself? (Evaluation?)
  - Do you prefer to treat certain illnesses or do you dislike treating others?
- What about the doctors' referral diagnosis? Do you often agree with them?
  - Would you like to have more prior knowledge about your patients?
- How satisfied are you with the education/ training courses?
- To what extent do patients' expectations influence your treatment?
- To what extent do you see yourself not only as a medical ‘service provider’, but also as a ‘helper’ in other areas? (In the sense of satisfying social indications)
  - How do patients react if you refuse the services they want?
- Do you feel restricted in your treatment by external circumstances? (time, money…)
- To what extent could you provide therapy differently without financial or time pressure?
- How satisfied are you with your working conditions? (Salary, time, etc.)
- Do you see any differences in the care of those with statutory insurance compared to those with private insurance?
- Can you think of any other reasons for overprovision?
- What problems can occur up to this point? Can you explain these using examples from your everyday life?
  - Specifically for patients?
  - For therapists?
  - For society as a whole?

1. **Strategies to reduce medical overuse**

Now I would like to discuss with you some approaches that can be used to reduce overuse

- What suggestions for solutions do you have?
- Who would you like to receive more support from?
  - Patient
  - Politics
  - Medical profession
  - Colleagues
- How could more awareness of the topic be created?
- Do you think regular and better training could limit the problem?
- Did the interview change your perspective? Why?
