# Supplementary material for: ‘Harm? I don’t think so!’: medical overuse from the perspective of allied health professionals in Germany – a qualitative study
Source: BMJ Open. 2025 Nov 4;15(11):e102991. doi: 10.1136/bmjopen-2025-102991 (PMC12587934; doi:10.1136/bmjopen-2025-102991)
Supplement: Supplementary file 2 [file bmjopen-2025-102991.reviewer_comments.pdf]

PEER REVIEW HISTORY

BMJ Open publishes all reviews undertaken for accepted manuscripts. Reviewers are asked to complete a checklist review form and are provided with free text boxes to elaborate on their assessment. These free text comments are reproduced below.

ARTICLE DETAILS

Title (Provisional)

“Harm? I don't think so!“ - Medical Overuse from the perspective of allied health professionals in Germany: A qualitative study

Authors

Stelzner, Benedikt; Rink, Laura; Kühlein, Thomas; Sebastiao, Maria

---

VERSION 1 - REVIEW

Reviewer 1

Name Elshaug, Adam

Affiliation The University of Melbourne School of Population and Global Health, Centre for Health Policy

Date 30-Jun-2025

COI None

Thank you for the opportunity to review this interesting paper an therapists understanding and perspectives of low-value care.

Initially I was concerned that the low n=14 would prove problematic for the rigour of any findings, however I note the authors report saturation, and furthermore while it might be most appropriate to view this investigation as somewhat of a pilot, the results are quite fascinating and illuminating of some important concepts in this area.

The authors have done a commendable job in clearly reporting the results section together with what I view as very reasonable and insightful interpretations. The insights provided certainly do contribute novel findings to this field which is dominated by medical profession insights.

I appreciate that the discussion introduces two or so policy recommendations (EBM training, guideline use, involvement of therapists in Choosing Wisely-type campaigns etc) however I would encourage a second list of 'future research priorities' in this area as it relates to therapists. Further, for bolstering communication I suggest creating two clear lsist, perhaps bullet pointed (or boxed) with the recommendations (both policy/practice and future research).

Some small items:

The term ‘therapists’ is used which i see as appropriate for the German context however ‘allied health professional’ or similar may be more appropriate for an international audience. The editor may have an opinion/suggestion on this.

I was unsure of why "[X]" is placed throughout the document? E.g.: Did this study have ethics approval? In the report it states committee of the [x] approved the study (file number x).

At line 48 (or 72) “…contacted therapists in [x]…”.

Forgive me if there is a blinding process here but I thought BMJ Open is now fully transparent, so I'm not sure if ethics was granted?

**Reviewer**	**2**
**Name**	**Arab-Zozani, Morteza**
**Affiliation**	**Birjand University of Medical Sciences, Health services management**
**Date**	**02-Aug-2025**
**COI**	**None**

Dear Respectable Authors

Thank you for considering a great area of research related to medical overuse and underuse. Your topic and results of interest to readers, but the way you conduct and report your manuscript needs some revisions as follows. I hope these comments will promote the quality of writing and reporting of your manuscript. Please respond to comments point-by-point and explain the changes for each comment in the main body of the manuscript.

Cheers

- Abstract, please mention the full term of the PTs, OTs, SLTs, and then mention the abbreviation. Also, add a brief about the therapist that you considered in your study. You can add this item in the main method of the manuscript.

- Abstract: A qualitative study is not a design. It is a general type of research. Please add what type of qualitative study you used here, like phenomenology, grounded theory, etc.

- Abstract, please add the sampling method and how you faced data saturation.

- You have selected three different groups of therapists. Each group's exposure to the topic of medical overuse may be different. In my opinion, the sample size selected does not adequately reflect the opinions of these broad spectrums, and for each group of therapists, at least this number of participants is needed to reach saturation. On the other hand, you have asked four different sets of questions, and with such a small number of samples, reaching data saturation in each question is a bit far-fetched.

- Your results in the abstract should be stated in the same order as the objectives, and at least the most important concepts obtained in each area of the questions should be mentioned.

- You have stated that you have examined Medical overuse through 4 questions, but you have also mentioned medical underuse in the results. How is this possible when the two issues are completely different and, in fact, opposite to each other?

- The conclusion is very brief, and it is interesting to note why the participants were selected if they had little knowledge of the study. In qualitative studies, we look for key informants; otherwise, our results will be far from reality, and perhaps this is the main reason for the general lack of results. Secondly, you have stated 4 main questions, and in the conclusion, you should briefly address all 4 issues.

- In the part of What this study adds, the items mentioned do not add much information to the existing body of knowledge. More precise results that are novel should be mentioned in this part.

- In the part of How this study might affect research, practice, or policy, you stated that Therapists can function as educators, but based on your results, and in my opinion, you first educate these therapists to enable them for future education. Also, how can we help tackle the problem of medical overuse? Please add practical recommendations, not general facts.

- Keywords, please add medical overuse as your main keywords.

- Background: The second paragraph is too long. Please break it into two or three paragraphs.

- You stated that you followed the COREQ checklist, but your method was not followed and addressed based on this checklist. Please structure based on the items of the checklist and mention all parts of the checklist in the methods section.

- You stated that: Transcripts were not provided to participants. Why and what is your reason? You missed an item that increases the rigor of a qualitative study. Please add the reason, and also add it to the limitations or weaknesses of the study.

- Please add a paragraph regarding the items that you used for increasing the rigor of the study. You missed this section in your methods section. Also, add the information regarding saturation and how to reach it.

- Please add an item about the description of participants and setting. The information you stated is not enough.

- What was the duration of the interview?

- In my opinion, the participants in your study were not key informants because about half of them were junior in their field with low years of experience and age.

- How do you justify this problem? Did you pay attention to this issue in the sampling and selection process? This is a major bias in sample selection. For example, the SLT03 participant

is a “ no idea” participant. In such a case, you need to exclude this participant from the interview and add new informed cases.

- In the theme of “Examples of medical overuse,” you need to categorize these items.

- Your aims are all about the overuse, but one main theme is about underuse: Underuse is a more urgent problem. This theme is completely unrelated to your study. Indeed, these two issues are completely intertwined, but they do not necessarily happen at the same time, and even the settings where they happen are different, and the same reasons cannot be given for the two. In addition, this issue contradicts the goals you mentioned.

- The drivers mentioned are too many and could be categorized more precisely. Almost all of these items have been mentioned many times in different studies. You should mention these drivers precisely from the therapists' point of view. It seems that the analysis of these parts is not done very well, and the analyst does not even have complete information about the subject and needs more training. Also, consequences and measures are not specifically related to the field, and such items are too general.

- The discussion is not well-formed. It is just a repetition of similar studies. In a qualitative work, there should be a comprehensive discussion considering specific results and comparison with similar studies, and finally ending with practical recommendations or Using such recommendations, practical solutions can be issued to reduce a problem or issue. The conclusion is quite general and needs substantial revision. Many limitations in your study have been mentioned in the comments, but have not been mentioned in this section.

VERSION 1 - AUTHOR RESPONSE

Reviewer: 1

Prof. Adam Elshaug, The University of Melbourne School of Population and Global Health
Comments to the Author:

Thank you for the opportunity to review this interesting paper on therapists understanding and perspectives of low-value care.

Initially I was concerned that the low n=14 would prove problematic for the rigour of any findings, however I note the authors report saturation, and furthermore while it might be most appropriate to view this investigation as somewhat of a pilot, the results are quite fascinating and illuminating of some important concepts in this area.

Our answer: Thank you very much for this positive feedback. We have included further information to back up our sampling. We also included in the manuscript now that we see our study as pilot.

Changes in the manuscript:
We see our study as a pilot to explore AHPs’ perspective on medical overuse.

At first, [Author 1] contacted AHPs in [X] randomly and informed them about the study via flyer and/or e-mail. AHPs could further spread the information. AHPs expressing interest subsequently received the study information, the data protection declaration, a consent form for signature, and a brief socio-demographic survey for completion. We planned to include not only one kind of AHP but PTs, OTs, and SLTs instead, to better capture system-level perceptions of medical overuse. Our aim was to capture how AHPs perceive medical overuse in routine practice, rather than to elicit only expert definitions. Inclusion of early-career AHPs was intentional as they constitute a substantial share of the German workforce and are most likely to have academic training (and exposure to EBP/guidelines). After the first interviews, recruitment was carried out iteratively, allowing for targeted engagement with specific professional groups following data collection and initial analysis. For example, the first three therapists interviewed had completed training, so we specifically looked for therapists with degrees. Another example is age of the interviewees: as the first interviewees were under 30 years of age, we thereafter sought out therapists who were older than 40 years as a contrast (interviews 6, 7, 8).

The authors have done a commendable job in clearly reporting the results section together with what I view as very reasonable and insightful interpretations. The insights provided certainly do contribute novel findings to this field which is dominated by medical profession insights.
Our answer: We highly appreciate this feedback.

I appreciate that the discussion introduces two or so policy recommendations (EBM training, guideline use, involvement of therapists in Choosing Wisely-type campaigns etc) however I would encourage a second list of 'future research priorities' in this area as it relates to therapists. Further, for bolstering communication I suggest creating two clear lists, perhaps bullet pointed (or boxed) with the recommendations (both policy/practice and future research).
Our answer: Thank you for this suggestion. We now included two lists.

Changes in the manuscript:
Implications for practice
- AHPs need further training to better understand the benefits and harms of their treatment.
- AHPs should be included in guideline development to ensure greater practicability.
- Change processes should begin where AHPs already show an emerging awareness of problems after study participation (e.g., fully using prescriptions even when it no longer necessary). However, changes should not result in income loss, in order to promote acceptance.

Implications for future research
- Based on our results a quantitative study with a focus on practical solutions may follow.
- Our study did not set out to examine medical underuse; results regarding underuse were participant-led and reported to contextualize perceptions of medical overuse. Future work should further investigate medical underuse with dedicated aims, sampling, and measures.

- To our knowledge, research primarily focuses on PTs. Future studies should also focus on OTs and SLTs.

Some small items: The term ‘therapists’ is used which i see as appropriate for the German context however ‘allied health professional’ or similar may be more appropriate for an international audience. The editor may have an opinion/suggestion on this.
Our answer: Thank you for your thoughts on this. We have changed the term and now use allied health professional.

I was unsure of why "[X]" is placed throughout the document? E.g.: Did this study have ethics approval? In the report it states committee of the [x] approved the study (file number x).
At line 48 (or 72) “…contacted therapists in [x]…”. Forgive me if there is a blinding process here but I thought BMJ Open is now fully transparent, so I'm not sure if ethics was granted?

Our answer: You are correct, we have inserted the x for anonymisation. The study was approved by the ethics committee. We do not report any clear data of participants but marked our names/ area of recruiting with an x in the manuscript as well. We will include this missing information after acceptance.

Reviewer: 2

Dr. Morteza Arab-Zozani, Birjand University of Medical Sciences
Comments to the Author:

Dear Respectable Authors
Thank you for considering a great area of research related to medical overuse and underuse. Your topic and results of interest to readers, but the way you conduct and report your manuscript needs some revisions as follows. I hope these comments will promote the quality of writing and reporting of your manuscript. Please respond to comments point-by-point and explain the changes for each comment in the main body of the manuscript.
Cheers

Abstract, please mention the full term of the PTs, OTs, SLTs, and then mention the abbreviation. Also, add a brief about the therapist that you considered in your study. You can add this item in the main method of the manuscript.

Our answer: We now use the full terms in the abstract and then the abbreviation throughout the text. Table 1 in the methods section lists further characteristics of the therapists that were included.

Changes in the manuscript (Abstract): 1) To what extent are physiotherapists (PT), occupational therapists (OT), or speech and language therapists (SLT) familiar with the phenomenon of medical overuse?

The previous interviews determined the selection of further participants. This meant that, by the end of the study, therapists were no longer approached at random, but were sought out specifically, in terms of profession and professional experience.

Abstract: A qualitative study is not a design. It is a general type of research. Please add what type of qualitative study you used here, like phenomenology, grounded theory, etc. Abstract, please add the sampling method and how you faced data saturation.

Our answer: A qualitative **descriptive** design was chosen to provide a practice-oriented understanding of how therapists perceive and handle medical overuse. This approach allows for a detailed description of participants' experiences. We added this information in the abstract and the methods.

Changes in the manuscript:
(Abstract)Design: This study used a qualitative descriptive design and applied qualitative content analysis to explore the therapists' point of view. A qualitative content analysis using a deductive-inductive approach was conducted. After coding half of the interviews, no further categories were added, indicating data saturation.

(Manuscript) [Author 1] conducted a qualitative content analysis using a deductive-inductive approach (with the software MAXQDA Plus 2020). Initially, categories were established based on the interview guide (perceptions of medical overuse, drivers, consequences of medical overuse, and measures to reduce overuse), which were then systematically applied to the data (deductive coding). The deductive frame comprised 21 subcategories in total (10 first-order and 11 second-order).
Throughout the analysis process, the category system was iteratively expanded through inductive coding (e.g., underuse, insufficient knowledge, therapist–patient relationship, lack of communication, ten new subcategories emerged). All categories were continuously discussed by the project team. We monitored data saturation continuously during coding.

You have selected three different groups of therapists. Each group's exposure to the topic of medical overuse may be different. In my opinion, the sample size selected does not adequately reflect the opinions of these broad spectrums, and for each group of therapists, at least this number of participants is needed to reach saturation. On the other hand, you have asked four different sets of questions, and with such a small number of samples, reaching data saturation in each question is a bit far-fetched.
Our answer: This study was primarily exploratory in nature, as the therapists' perspective has not yet been examined in Germany. We therefore made a conscious decision to include all three therapeutic professions. We consider this broad perspective to be appropriate for an initial exploration and approach to the topic. It would have been possible that the therapists had very different perceptions of the topic. In this case we would have agreed that the number would not have been sufficient. However, this was not the case. The perceptions of the topic were fairly homogenous. This is why we did not expect any new information after fourteen interviews and reached saturation. We also want to note: A common characteristic of these three professional groups is their dependence on medical referral or indication, which plays a crucial role in discussions on potential overuse within the healthcare system. In this regard, the professional background plays only a subordinate role. We therefore respectfully ask to accept that our sample was sufficient for data saturation.
As in other studies, it became apparent that medical overuse was relatively unknown also in this group of therapists. What we observed is that it was easier for participants (regardless of their profession) to name medical overuse in physiotherapy. This explains the differences in

sample size across the professions. In speech therapy, no substantially new insights emerged after the third interview. We now emphasized more explicitly the exploratory nature of our study and further elaborated the problem you raised in the limitations section.
We have also listed implications for the future in the discussion. Here, we now discuss future studies and further possibilities for differentiation.

Changes in the manuscript:
Limitations
Our goal was to explore the AHPs' perspective on medical overuse. We deliberately did not exclude the participants that were unfamiliar with medical overuse, as this is a key finding and is consistent with findings from other professions. Across professions, answers were largely homogeneous. Unequal numbers in the sample across professions reflect saturation rather than bias—SLT yielded no substantially new insights after interview three, whereas additional PT interviews added nuance. Given the shared dependency on medical referral across these professions, common system drivers outweighed profession-specific differences.

Implications for practice
- AHPs need further training to better understand the benefits and harms of their treatment.
- AHPs should be included in guideline development to ensure greater practicability.
- Change processes should begin where AHPs already show an emerging awareness of problems after study participation (e.g., fully using prescriptions even when it no longer necessary). However, changes should not result in income loss, in order to promote acceptance.
-

Implications for future research
- Based on our results a quantitative study may follow and focus on practical solutions.
- Our study did not set out to examine medical underuse; results regarding underuse were participant-led and reported to contextualize perceptions of medical overuse. Future work should further investigate medical underuse with dedicated aims, sampling, and measures.
- To our knowledge, research primarily focuses on PTs. Future studies should also focus on OTs and SLTs.

Your results in the abstract should be stated in the same order as the objectives, and at least the most important concepts obtained in each area of the questions should be mentioned.

Our answer: Thank you for this feedback, you are right and we rearranged the results section of the abstract.
[Research Question 1) To what extent are physiotherapists (PT), occupational therapists (OT), or speech and language therapists (SLT) familiar with the phenomenon of medical overuse?]
AHPs struggled to define overuse. To them, underuse was perceived as a much more pressing issue.
[Research Question 2) What drivers do PTs, OTs and SLTs suspect?]
AHPs identified structural, economic, physician and patient-driven factors. They did not see themselves as part of the problem of medical overuse
[Research Question 3) What are the consequences of medical overuse?]
and assumed that their treatment, even without indication, has little to no disadvantage for patients.

[Research Question 4) What measures can be taken to reduce medical overuse?]
AHPs found it difficult to derive specific solutions, they named terminating unnecessary therapies and healthcare system reforms.

Changes in the manuscript:
AHPs struggled to define overuse. To them, underuse was perceived as a much more pressing issue. AHPs identified structural, economic, physician and patient-driven factors. They did not see themselves as part of the problem of medical overuse and assumed that their treatment, even without indication, has little to no disadvantage for patients. AHPs found it difficult to derive specific solutions, they named terminating unnecessary therapies and healthcare system reforms.

You have stated that you have examined Medical overuse through 4 questions, but you have also mentioned medical underuse in the results. How is this possible when the two issues are completely different and, in fact, opposite to each other?

Our answer: We agree that medical overuse and underuse are distinct and even opposite concepts. However, they can not only take place in parallel at the same time but overuse might even be the cause of underuse for example by occupying work force urgently needed elsewhere. In our study medical underuse emerged as an issue from the side of the interviewees, although it was not part of our original research questions. Specifically, when asked about medical overuse, many therapists spontaneously shifted the conversation towards medical underuse. They described long waiting lists, a shortage of therapists, and difficulties in obtaining therapy prescriptions as the much more pressing problem in their daily practice. This indicates that, for therapists, medical underuse represents the dominant lived experience, while medical overuse is less familiar and less frequently reflected upon. And, as we pointed out, there was little self-reflection on therapists' induced underuse due to medical overuse elsewhere e.g. due too long treatment exceeding the needs of single patients.
While the interview guide was semi-structured around four pre-defined research questions on medical overuse, the inductive part of the content analysis allowed further themes to emerge (in this cases medical underuse). We reported this theme as its strong presence in the data reflects how therapists themselves perceive the balance of problems in the healthcare system and contextualizes the therapists' understanding (or lack thereof) of medical overuse, while at the same time our main focus remained on medical overuse.

Changes in the manuscript:
Discussion:
Participants' focus on underuse (e.g., long waiting lists) helps explain why AHPs perceived medical overuse as less immediate and, at times, harder to recognize.

The study did not set out to examine medical underuse. However, the qualitative descriptive study design allowed for new topics to emerge such as underuse. These results were participant-led and reported to contextualize perceptions of medical overuse.

The conclusion is very brief, and it is interesting to note why the participants were selected if they had little knowledge of the study. In qualitative studies, we look for key informants; otherwise, our results will be far from reality, and perhaps this is the main reason for the

general lack of results. Secondly, you have stated 4 main questions, and in the conclusion, you should briefly address all 4 issues.

Our answer: We kindly disagree with you here and would like to explain our reasoning. The aim of our study was, on the one hand, to gather expert opinions on the term medical overuse, but on the other also to explore how ordinary physiotherapists, occupational therapists, and speech therapists perceive, understand, and make sense of the concept in their everyday practice. The lack of awareness and difficulties in defining medical overuse therefore represent a key result, not a limitation of our sampling. In fact, this gap in knowledge reflects the reality that medical overuse has so far been largely discussed in the context of physicians, while therapists' perspectives have rarely been considered. And even among physicians, studies came to a similar result that medical overuse is rather unknown.
By including therapists regardless of their prior familiarity with the topic, we were able to reveal, that they see themselves as having little influence on medical overuse and that education will be necessary to involve therapists in future initiatives to reduce medical overuse. We could also show that despite not knowing medical overuse beforehand they could think about and engage with the topic. We believe, that the limited knowledge of the participants therefore does not indicate a lack of results, but rather underscores an important finding of our study: Medical overuse is not yet an established concept among therapists in Germany. However, as we believe that other readers may have the same concern as you, we now included your thoughts in the method and discussion. Please see our changes mentioned above. Regarding your second remark, we are now addressing all 4 research questions in the conclusion (to a different level of detail). As reviewer 1 recommended bullet points for the implication, we added those as well.

Changes in the manuscript:
[knowledge]
Our results showed on the one hand that AHPs were rather unfamiliar with the concept of medical overuse, suggesting that more awareness raising is needed. On the other hand, the AHPs developed an understanding for the issue, showing that asking or better education can trigger reflection. The participants found it easier to name medical overuse within PT (e.g., massages).
[driver]
AHPs saw the main driver of medical overuse in physicians and patients' demands. Because they depend on physicians' prescriptions, AHPs may perceive themselves as less responsible for medical overuse.
[consequences]
They did not see any harm due to treatment.
[solutions]

Implications for practice
- AHPs need further training to better understand the benefits and harms of their treatment.
- AHPs should be included in guideline development to ensure greater practicability.
- Change processes should begin where AHPs already show an emerging awareness of problems after study participation (e.g., fully using prescriptions even when it no longer necessary). However, changes should not result in income loss, in order to promote acceptance.

Implications for future research

- Based on our results a quantitative study with a focus on practical solutions may follow.
- Our study did not set out to examine medical underuse; results regarding underuse were participant-led and reported to contextualize perceptions of medical overuse. Future work should further investigate medical underuse with dedicated aims, sampling, and measures.
- To our knowledge, research primarily focuses on PTs. Future studies should also focus on OTs and SLTs.

In the part of what this study adds, the items mentioned do not add much information to the existing body of knowledge. More precise results that are novel should be mentioned in this part.

Our answer: To our knowledge, this is the first study that explores the therapists' point of view and therefore our results do add to the existing literature.

In the part of How this study might affect research, practice, or policy, you stated that Therapists can function as educators, but based on your results, and in my opinion, you first educate these therapists to enable them for future education. Also, how can we help tackle the problem of medical overuse? Please add practical recommendations, not general facts.
Our answer: You are absolutely right, therapists are (one of the first) who need to be educated themselves. Only in a second step, they could be perfect as multiplicators and educators as they have more time with patients than physicians mostly have. As a concrete step, practical guidelines are needed, along with awareness of their existence and their use in practice. To this end, more therapists are gradually being appointed to guideline commissions in Germany, and this development should be further expanded and reinforced. We included further information in den discussion. Here, we focus on the demand for direct access.

Changes in the manuscript:
How this study might affect research, practice or policy: The lack of knowledge about medical overuse demonstrates the importance of sensitizing AHPs for the issue so that they can educate patients. AHPs in Germany wish for direct access which requires increased responsibility for economic efficiency and a solid understanding of evidence-based-therapy.

Keywords, please add medical overuse as your main keywords.
Our answer: Thank you, for pointing this out, however, we could not include own keywords.

Background: The second paragraph is too long. Please break it into two or three paragraphs.
Our answer: Thank you for pointing this out, we broke it down.

You stated that you followed the COREQ checklist, but your method was not followed and addressed based on this checklist. Please structure based on the items of the checklist and mention all parts of the checklist in the methods section.
Our answer: We have used the checklist and addressed all points in the manuscript. You can find the checklist with our answers at the end of this point-by-point.

You stated that: Transcripts were not provided to participants. Why and what is your reason? You missed an item that increases the rigor of a qualitative study. Please add the reason, and also add it to the limitations or weaknesses of the study.

Our answer: We did not provide the transcripts to participants during the interviews as subsequent comments or corrections by participants may alter the original data collection. Participants did not receive any kind of incentives, so another reason why we did not provide the transcripts to participants was time and resource-efficiency. However, we asked about interest in the results and documented possible interests.

Changes in the manuscript: No changes were made.

Please add a paragraph regarding the items that you used for increasing the rigor of the study. You missed this section in your methods section. Also, add the information regarding saturation and how to reach it.

Our answer: We included a new section 'Quality assurance' in which we included further information.

Changes in the manuscript:

We took multiple steps to assure the quality of this study: the interviewer received training in qualitative interviewing by two senior researchers(author 2 and author 4), the interview guide was piloted with two AHPs and refined accordingly, data triangulation took place through interviews with different AHPs (heterogeneity across profession, age, gender, qualification, and setting), after each interview field notes were made (e.g. disturbances during interview, first ideas for further recruitment and/or data analysis), the research diary was not only kept the entire research process, but also repeatedly incorporated into data collection and analysis. To strengthen dependability and confirmability, [author 4] reviewed coding, and discrepancies were discussed to consensus. We also conducted a qualitative research workshop to code and discuss codes with other researchers.

Please add an item about the description of participants and setting. The information you stated is not enough.

Our answer: We included further information about the recruitment process and details on participants.

Changes in the manuscript:

We planned to include not only one kind of AHP but PTs, OTs, and SLTs instead, to better capture system-level perceptions of medical overuse. Our aim was to capture how AHPs perceive medical overuse in routine practice, rather than to elicit only expert definitions. Inclusion of early-career AHPs was intentional as they constitute a substantial share of the German workforce and are most likely to have academic training (and exposure to EBP/guidelines). After the first interviews, recruitment was carried out iteratively, allowing for targeted engagement with specific professional groups following data collection and initial analysis. For example, the first three therapists interviewed had completed training, so we specifically looked for therapists with degrees. Another example is age of the interviewees: as the first interviewees were under 30 years of age, we thereafter sought out therapists who were older than 40 years as a contrast (interviews 6, 7, 8).

What was the duration of the interview?

Our answer: The interviews lasted an average of 54 minutes. The shortest was 40 minutes, the longest 60 minutes. We included this information in the manuscript.

Changes in the manuscript: [Author 1] conducted all interviews (n = 14, between 40 - 60 minutes, average 54 minutes in length) via video calls using the software Zoom.

In my opinion, the participants in your study were not key informants because about half of them were junior in their field with low years of experience and age.

Our answer: We acknowledge that many of our participants were relatively junior in their profession. Our aim was not to recruit key informants regarding medical overuse, but rather to explore how therapists with different backgrounds, ages, and levels of experience perceive medical overuse. Including younger therapists was important for several reasons. They represent a substantial proportion of the current workforce. There is a high rate of transition to other professions in the therapeutic professions in Germany, meaning that therapists tend to be younger. We assumed that younger therapists would be more familiar with the concept. A limitation we have mentioned in the manuscript is that we were not able to include therapists with a Master's or doctorate degree.
Academicisation of therapists has not progressed very far in Germany, and it is primarily young therapists who have academic degrees, so there was a higher chance that the concept would be familiar here. Despite this, their lack of familiarity with the concept itself is a key finding, showing that medical overuse has not yet been integrated into training. It also shows that the training of therapists lacks working with guidelines, as otherwise especially young participants would have to know the concepts.

Changes in the manuscript:
Inclusion of early-career therapists was intentional. They constitute a substantial share of the German workforce and are most likely to have academic training (and exposure to EBP/guidelines). Our aim was to capture how therapists at different stages perceive and handle medical overuse in routine practice, rather than to elicit only expert definitions.

How do you justify this problem? Did you pay attention to this issue in the sampling and selection process? This is a major bias in sample selection. For example, the SLT03 participant is a "no idea" participant. In such a case, you need to exclude this participant from the interview and add new informed cases.

Our answer: As we tried to explain in the paragraph above, our goal was not to interview key informants per se but to broadly explore the therapists' perspective. We deliberately did not exclude the participants, as not being familiar with medical overuse is a key finding and is consistent with findings from other professions. Methodologically, we do not differ from other studies in this respect. This aspect is also discussed in the limitations.

Changes in the manuscript:

We planned to include not only one kind of AHP but PTs, OTs, and SLTs instead, to better capture system-level perceptions of medical overuse. Our aim was to capture how AHPs perceive medical overuse in routine practice, rather than to elicit only expert definitions.
Our goal was to explore the AHPs' perspective on medical overuse. We deliberately did not exclude the participants that were unfamiliar with medical overuse, as this is a key finding and is consistent with findings from other professions. Across professions, answers were largely homogeneous. Unequal numbers in the sample across professions reflect saturation rather than bias—SLT yielded no substantially new insights after interview three, whereas additional PT interviews added nuance. Given the shared dependency on medical referral across these professions, common system drivers outweighed profession-specific differences.

In the theme of "Examples of medical overuse," you need to categorize these items.

Our answer: During data analysis we formed three categories for examples: 1) within profession, 2) within other allied health care professions, and 3) other professions. The examples we give, come from these groups. We have made the categories clearer in terms of language. However, as there were not many examples overall, we were unable to create any further categories.

Changes in the manuscript:

The AHPs were able to mention specific unnecessary treatments. However, it was noticeable that this only became possible during the course of the interview, after a certain level of engagement with the topic had already occurred. Examples of medical overuse were 1) within their profession ("*Thermal treatments [...] that's absolutely pointless*." (OT04)). Especially in SLT, it was challenging for participants to name examples: "*This is the only area that comes to mind […] children with bilingualism. First of all, to differentiate, does the child need therapy or language support? That's a fine line.*" (SLT02). 2) within other allied health care professions (e.g., massages for certain problems), and 3) other professions/ medical areas. Here, AHPs found it easier to provide examples, such as surgeries, or excessive prescription of medications (e.g., antibiotic administration for flu-like infections or polypharmacy in older individuals) (PT03).

Your aims are all about the overuse, but one main theme is about underuse: Underuse is a more urgent problem. This theme is completely unrelated to your study. Indeed, these two issues are completely intertwined, but they do not necessarily happen at the same time, and even the settings where they happen are different, and the same reasons cannot be given for the two. In addition, this issue contradicts the goals you mentioned.

Our answer: You are are correct, our aim was to focus on medical overuse. In the interviews many therapists spontaneously contrasted medical overuse with underuse (e.g., long waiting lists, insufficient prescriptions, workforce shortages) and framed underuse as the more pressing day-to-day problem. We reported this as an emergent, contextual theme because it helps explaining central findings: 1) Therapists low familiarity with the concept of medical overuse, 2) emphasizes that they see themselves as having little influence and 3) their difficulty naming solutions for medical overuse.

Our results also show that there is little self-reflection, for example, on the fact that in some cases underuse may also be caused by therapists themselves, e.g. by performing therapy for longer than necessary or providing therapy not supported by evidence. We therefore see the need to present medical overuse and underuse together.

Changes in the manuscript:

Results

In the interviews many AHPs spontaneously contrasted medical overuse with underuse (e.g., long waiting lists, insufficient prescriptions, workforce shortages) and framed underuse as the more pressing day-to-day problem: *"Everyone could not only double themselves but triple, and we would still have plenty to do*." (OT01). For them, patients also receive too few prescriptions for therapy: *"You just have to say that you have to kind of fight for prescriptions for therapy" (SLT02).*

Discussion

Participants' focus on underuse (e.g., long waiting lists) helps explain why AHPs perceived medical overuse as less immediate and, at times, harder to recognize.

The study did not set out to examine medical underuse; results regarding underuse were participant-led and reported to contextualize perceptions of medical overuse. Future work should further investigate medical underuse with dedicated aims, sampling, and measures.

The drivers mentioned are too many and could be categorized more precisely. Almost all of these items have been mentioned many times in different studies. You should mention these drivers precisely from the therapists' point of view. It seems that the analysis of these parts is not done very well, and the analyst does not even have complete information about the subject and needs more training. Also, consequences and measures are not specifically related to the field, and such items are too general.

Our answer: Author 1 received adequate training before data collection and was closely supervised during data analysis which we now describe in a new section on quality assurance (see also our answer above). The drivers mentioned in the manuscript are the ones mentioned by the therapists and as you pointed out, they do not differ very much from other studies. It is true that certain influencing factors, such as physicians, are mentioned even though they may not appear directly connected to the therapists at first glance. However, all aspects presented—such as cooperation or physicians' limited knowledge about the therapy—are closely linked to the therapists' professional context. The superficial nature of these points is not a result of shortcomings in the analysis, but rather due to the vague or limited responses given by the participants.
In response to your valuable feedback, we deleted larges parts of the drivers and included a figure. We believe that the results are more clearly presented and quicker to comprehend. We hope that this works better for you.

Changes in the manuscript:

AHPs mentioned drivers of medical overuse and identified physicians as the main driver of medical overuse but not concerning PT, OT or SLT, where the AHPs described only few cases in which physicians unnecessarily prescribed therapy (PT02) (**Fig.1**). In their opinion, physicians drive medical overuse in their own field. In general, AHPs did not see themselves

as part of the problem of medical overuse. However, during the course of the interviews, some respondents reflected more on their role, e.g. whether the problem of waiting lists is self-inflicted, as patients continue to be treated despite questionable effects (PT03). However, they described little to no action taken to refrain from treating patients to free up resources.

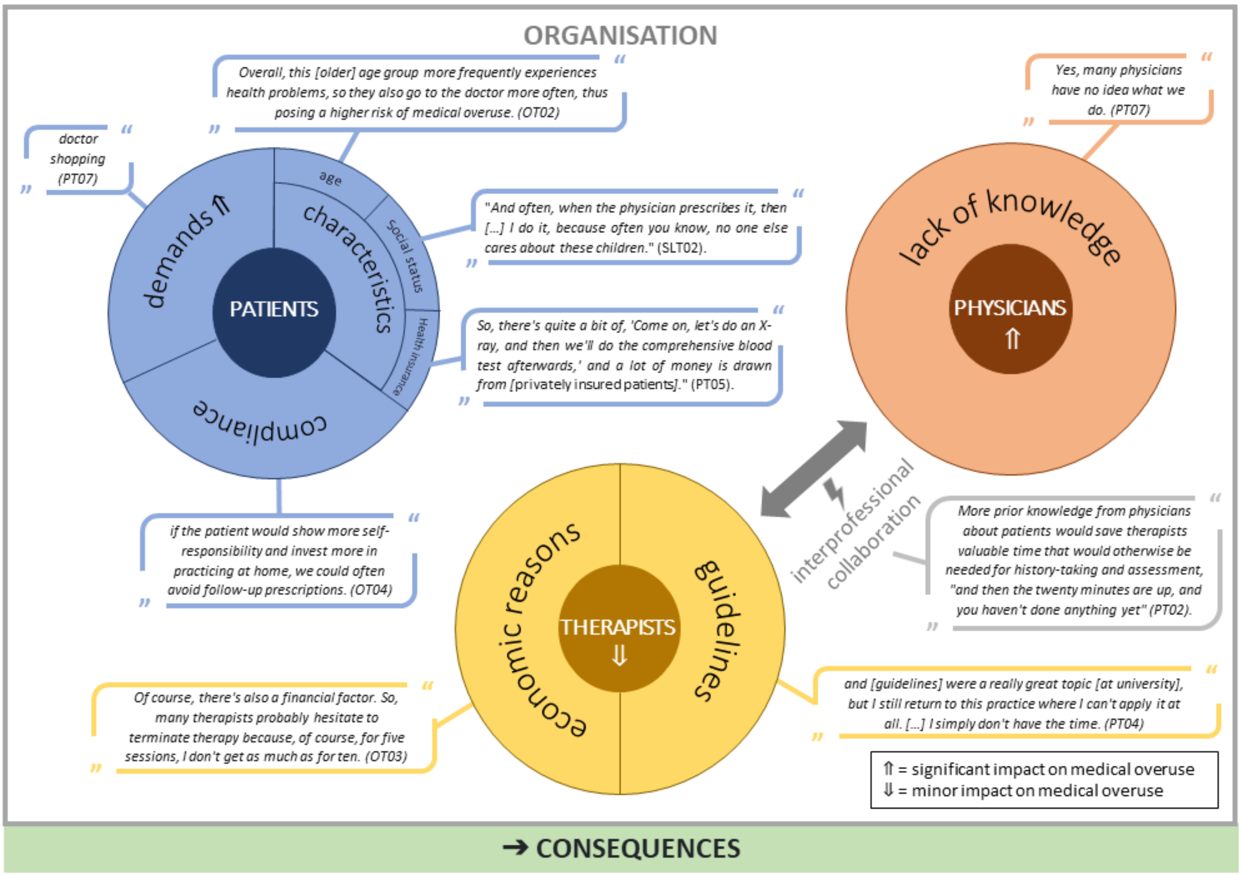

Figure 1. Drivers of medical overuse

The discussion is not well-formed. It is just a repetition of similar studies. In a qualitative work, there should be a comprehensive discussion considering specific results and comparison with similar studies, and finally ending with practical recommendations or Using such recommendations, practical solutions can be issued to reduce a problem or issue. The conclusion is quite general and needs substantial revision.

Our answer: Thank you for your feedback on the discussion. We have restructured the discussion and are now discussing the individual results one after the other. In our discussion, we consider other studies on medical overuse from Germany and compare our results. We are convinced that confirmation of existing results from this new perspective strengthens research on medical overuse.
We also provide practical recommendations (e.g. education, practical guidelines).
Please find our changes below, highlights in green will be deleted and are only for your information.

Changes in the manuscript:

[*Knowledge*]
The lack of knowledge about medical overuse was also evident in other studies (14, 25). Participants' focus on underuse (e.g., long waiting lists) helps explain why AHPs perceived medical overuse as less immediate and, at times, harder to recognize.
[Drivers]
The dependency on the prescription from physicians could explain why AHPs saw themselves as having little responsibility for medical overuse. AHPs saw the main driver of medical overuse in physicians. Patients' demands seem to be another critical driver of medical overuse which was stated in previous studies (14, 15, 26, 27). It was not clear whether the patients always verbalize these demands or whether they are assumed by physicians/therapists. Other studies have shown that physicians sometimes act based on assumed demands (28). The phenomenon of blaming others could also be found in physicians and patients (14, 16). AHPs also believed that contact with the healthcare system itself already raises the risk of medical overuse which was also mentioned by patients in Germany (14). Routines are another factor that drives medical overuse as they do not require a conscious decision-making (29, 30). This point was not mentioned directly by the AHPs, but it became apparent that the AHPs rarely deviated from the treatment plan, for example by ending the treatment earlier than prescribed.
[*Consequences]*
AHPs saw little to no harm in therapy even without indication. It can be assumed that AHPs, like patients and physicians (31, 32), overestimate the benefits and underestimate the harms of interventions.
[*Solution]*
Studies have shown incorrect beliefs about low back pain treatment among PTs (34, 35). In an international review between 48 % and 70 % of PTs could critically appraise research papers (33). Training in evidence-based therapy may vary greatly in Germany due to different training paths. It is possible to start treating patients after a 3-year vocational training or to continue studying at universities of applied health sciences to receive a Bachelor's or Master's degree. A survey of 1,361 German physiotherapists revealed that only one third were familiar with the national guideline on unspecific low back pain. Longer professional experience could have a negative impact on treatment choices (36). Academically trained PTs perform slightly better in diagnostics (37), but the overall quality of decision-making remains below the ideal level. Learning to understand what evidence is and how it should be applied in practice should be trained intensively during all training stages. The lack of knowledge about medical overuse demonstrates the importance of sensitizing AHPs for the issue so that they can inform and educate patients in the long run —particularly as they often have more time with patients than physicians. However, further training was not mentioned by the AHPs as a solution.
Overall, proposed solutions were pragmatic but sparse: improving interprofessional communication which was also wished for by German GPs (26), and system-level reforms. A complete electronic medical record - which was introduced for all statutory health insured patients in Germany in 2025 - might support this need.
In 2024, a new regulation was introduced for selected indications, allowing OTs and PTs to independently determine the type and amount of treatment provided („open prescription"). With this change, financial responsibility was also transferred to the therapists: beyond a certain number of therapy sessions, financial deductions must be accepted. This regulation is not equivalent to direct access as in other countries which was mentioned as a solution. However, it grants AHPs more therapeutic freedom and responsibility. The impact of this regulation on existing care shortages and therapists' role perceptions is yet to be determined.

In other studies, direct access to physical therapy showed promising results on managing musculoskeletal disorders regarding for instance health care costs (36-38).
Clear recommendations (e.g., Choosing Wisely-style lists) were viewed favourably. An example could be the living guideline "*Protection against the Overuse and Underuse of Health Care*" published The German College of General Practitioners and Family Physicians (DEGAM) which compiles recommendations for protection against over- and underuse. A survey among German GPs showed that the majority of GPs found the guideline helpful and wished for further recommendations (26). Choosing Wisely Brazil for example developed a top 5 low-value-list for musculoskeletal physical therapy (39). Kharel et al. showed that PTs were more willing to follow detailed and positively framed recommendations or if evidence-based alternatives are given (40). These findings can help formulating recommendation for German AHPs as well. However, lists of recommendations on their own are unlikely to change practice (21) as a wide range of system-drivers promote medical overuse. This was also seen by AHPs who wished for changes of the reimbursement system. They rated false financial incentives as an important driver of medical overuse which is in line with previous studies (16).

Many limitations in your study have been mentioned in the comments, but have not been mentioned in this section.

Our answer: We have included further thoughts in this section, please see also our answers above.

Changes in the manuscript: AHPs voluntarily and without remuneration participated in the present study which may have led to a selection bias. Despite the small sample, we were able to recruit AHPs from all three disciplines. However, we were not able to include AHPs with a Master's or doctorate degree. We see our study as a pilot to explore AHPs' perspective on medical overuse. We deliberately did not exclude the participants that were unfamiliar with medical overuse, as this is a key finding and is consistent with findings from other professions. Across professions, answers were largely homogeneous. Unequal numbers in the sample across professions reflect saturation rather than bias — SLT yielded no substantially new insights after interview three, whereas additional PT interviews added nuance. Given the shared dependency on medical referral across these professions, common system drivers outweighed profession-specific differences. The study did not set out to examine medical underuse. However, the qualitative descriptive study design allowed for new topics to emerge such as underuse. These results were participant-led and reported to contextualize perceptions of medical overuse.

COREQ (COnsolidated criteria for REporting Qualitative research) Checklist

Topic	Item No.	Guide Questions/Description	Reported on Page No. (clean document)	Text passage
**Domain 1: Research team and reflexivity**				
Personal characteristics				
Interviewer/facilitator	1	Which author/s conducted the interview or focus group?	5	The interviewer ([Author 1], male, student of human medicine)
Credentials	2	What were the researcher's credentials? e.g. PhD, MD	5<br><br><br>6	The interviewer ([Author 1], male, student of human medicine)<br><br>[Author 1] was trained in qualitative data collection by [Author 3] (male, Chair of Institute, GP) and [Author 4] (female, senior researcher, SLT/Public Health).
Occupation	3	What was their occupation at the time of the study?	5,6	Please see above
Gender	4	Was the researcher male or female?	5,6	Please see above
Experience and training	5	What experience or training did the researcher have?	6<br><br><br>7	[Author 1] was trained in qualitative data collection by [Author 3] (male, Chair of Institute, GP) and [Author 4] (female, senior researcher, SLT/Public Health). the interviewer had training in qualitative interviewing by two senior researchers ([author 2 and author 4])<br>We also conducted a qualitative research workshop to code and discuss codes with other researchers
Relationship with participants				

Relationship established	6	Was a relationship established prior to study commencement?	5	The interviewer ([Author 1], male, student of human medicine) and the AHPs therapists did not know each other beforehand
Participant knowledge of the interviewer	7	What did the participants know about the researcher? e.g. personal goals, reasons for doing the research	5	Participants were informed about the goal of the study and gave written informed consent.
Interviewer characteristics	8	What characteristics were reported about the interviewer/facilitator? e.g. bias, assumptions, reasons and interests in the research topic	5<br><br>declarations	Our aim was to capture how AHPs perceive medical overuse in routine practice, rather than to elicit only expert definitions. Inclusion of early-career AHPs was intentional as they constitute a substantial share of the German workforce and are most likely to have academic training (and exposure to EBP/guidelines).<br><br>the research diary was not only kept the entire research process, but also repeatedly incorporated into data collection and analysis.<br><br>**Authors' information**<br>The present work was performed in (partial) fulfillment of the requirements for obtaining the degree „Dr. med.“ for [Author 1] at the [X].
**Domain 2: Study design**				
Theoretical framework				

Methodological orientation and Theory	9	What methodological orientation was stated to underpin the study? e.g. grounded theory, discourse analysis, ethnography, phenomenology, content analysis	4	This study used a qualitative descriptive design and applied qualitative content analysis to explore the AHPs' point of view qualitative content analysis using a deductive-inductive approach
Participant selection				
Sampling	10	How were participants selected? e.g. purposive, convenience, consecutive, snowball	4	[Author 1] contacted AHPs therapists in [X] randomly<br>AHPs Therapists could further spread the information
Method of approach	11	How were participants approached? e.g. face-to-face, telephone, mail, email	4f.	At first, [Author 1] contacted AHPs in [X] randomly and informed them about the study via flyer and/or e-mail. AHPs could further spread the information. AHPs expressing interest subsequently received the study information, the data protection declaration, a consent form for signature, and a brief socio-demographic survey for completion.
Sample size	12	How many participants were in the study?	8	Of the fourteen AHPs interviewed, most were female (n = 10) and were employed (Table 1).
Non-participation	13	How many people refused to participate or dropped out? Reasons?	5	None<br>All interested AHPs could take part in the study.
Setting				
Setting of data collection	14	Where was the data collected? e.g. home, clinic, workplace	6	video calls using the software Zoom
Presence of non-participants	15	Was anyone else present besides the participants and researchers?	6	[Author 1] conducted all interviews (n = 14, between 40 - 60 minutes, average 54

				minutes in length) via video calls using the software Zoom. Except for the participants, there was no one else present during data collection.
Description of sample	16	What are the important characteristics of the sample? e.g. demographic data, date		Table 1
Data collection				
Interview guide	17	Were questions, prompts, guides provided by the authors? Was it pilot tested?	5f	The project team developed the interview guide The interview guide underwent a pre-testing phase involving two AHPs the interview guide was piloted with two AHPs and refined accordingly
Repeat interviews	18	Were repeat interviews carried out? If yes, how many?	6	Interviews did not need to be repeated.
Audio/visual recording	19	Did the research use audio or visual recording to collect the data?	6	The sessions were digitally recorded, subsequently transcribed verbatim and pseudonymized using f4 software
Field notes	20	Were field notes made during and/or after the interview or focus group?	7	after each interview field notes were made (e.g. disturbances during interview, first ideas for data collection and/or further recruitment)
Duration	21	What was the duration of the interview or focus group?	6	n = 14, between 40 - 60 minutes, average 54 minutes in length
Data saturation	22	Was data saturation discussed?	6	We monitored data saturation continuously during coding After coding half of the interviews, no further categories were added, indicating data saturation

Transcripts returned	23	Were transcripts returned to participants for comment and/or correction?	6	Transcripts were not provided to participants.
**Domain 3: analysis and findings**				
Data analysis				
Number of data coders	24	How many data coders coded the data?	6f.	[Author 1] conducted a qualitative content analysis using a deductive-inductive approach (with the software MAXQDA Plus 2020).<br>To strengthen dependability and confirmability, [author 4] reviewed coding, and discrepancies were discussed to consensus. We also conducted a qualitative research workshop to code and discuss codes with other researchers.
Description of the coding tree	25	Did authors provide a description of the coding tree?		Initially, categories were established based on the interview guide (perceptions of medical overuse, drivers, consequences of medical overuse, and measures to reduce overuse), which were then systematically applied to the data (deductive coding). The deductive frame comprised 21 subcategories in total (10 first-order and 11 second-order).<br>Throughout the analysis process, the category system was iteratively expanded through inductive coding (e.g., underuse, insufficient knowledge, therapist–patient relationship,

				lack of communication; ten new subcategories emerged).
Derivation of themes	26	Were themes identified in advance or derived from the data?	6	Initially, categories were established based on the interview guide, which were then systematically applied to the data (deductive coding)<br>Throughout the analysis process, the category system was iteratively expanded through inductive coding
Software	27	What software, if applicable, was used to manage the data?	6	with the software MAXQDA Plus 2020
Participant checking	28	Did participants provide feedback on the findings?	6	Participants did not provide feedback on the findings
Reporting				
Quotations presented	29	Were participant quotations presented to illustrate the themes/findings? Was each quotation identified? e.g. participant number	Results	"*So, I haven't really dealt with the term. [...] No idea*" (SLT03) etc.
Data and findings consistent	30	Was there consistency between the data presented and the findings?	Results	Yes, please see the results section
Clarity of major themes	31	Were major themes clearly presented in the findings?	Results	Therapists were not familiar with medical overuse<br>Underuse is a more urgent problem<br>Drivers<br>Organisational structures<br>Consequences of medical overuse<br>Measures to reduce medical overuse
Clarity of minor themes	32	Is there a description of diverse cases or	Results	Yes, e.g. the examples were a minor theme

		discussion of minor themes?		and differed between participants.

Developed from: Tong A, Sainsbury P, Craig J. Consolidated criteria for reporting qualitative research (COREQ): a 32-item checklist for interviews and focus groups. *International Journal for Quality in Health Care*. 2007. Volume 19, Number 6: pp. 349 – 357
